# Increased Responsiveness to Toll-Like Receptor 4 Stimulation in Peripheral Blood Mononuclear Cells from Patients with Recent Onset Rheumatoid Arthritis

**DOI:** 10.1155/2008/132732

**Published:** 2008-06-23

**Authors:** M. L. Kowalski, A. Wolska, J. Grzegorczyk, J. Hilt, M. Jarzebska, M. Drobniewski, M. Synder, M. Kurowski

**Affiliations:** ^1^Department of Immunology, Rheumatology and Allergy, Chair of Immunology, Medical University of Lodz, Pomorska 251, 92-213 Lodz, Poland; ^2^Department of Laboratory Medical Immunology, Medical University of Lodz, Pomorska 251, 92-213 Lodz, Poland; ^3^Department of Orthopedics and Pediatric Orthopedics, Medical University of Lodz, Drewnowska 75, 91-002 Lodz, Poland

## Abstract

*Background*. Cell signaling via Toll-like receptors (TLRs) leads to synovial inflammation in rheumatoid arthritis (RA). We aimed to assess effects of TLR2 and TLR4 stimulation on proinflammatory cytokine production by peripheral blood mononuclear cells (PBMCs) from patients with recent-onset RA, osteoarthrosis (OA), and healthy control (HC). *Methods*. PBMCs were stimulated with LPS, biglycan and cytokine mix. Cytokines were analyzed in supernatants with ELISA. Expression of toll-like receptors mRNA in leukocytes was analyzed using real-time qPCR. *Results*. PBMCs from RA patients spontaneously produced less IL-6 and TNF*α* than cells from OA and HC subjects. LPS increased cytokines' production in all groups. In RA patients increase was dramatic (30 to 48-fold and 17 to 31-fold, for respective cytokines) compared to moderate (2 to 8-fold) in other groups. LPS induced 15-HETE generation in PBMCs from RA (mean 251%) and OA patients (mean 43%), although only in OA group, the increase was significant. TLR2 and TLR4 gene expressions decreased in response to cytokine mix, while LPS enhanced TLR2 expression in HC and depressed TLR4 expression in OA patients. *Conclusion*. PBMCs from recent-onset RA patients are overresponsive to stimulation with bacterial lipopolysaccharide. TLR expression is differentially regulated in healthy and arthritic subjects.

## 1. INTRODUCTION

Rheumatoid arthritis (RA) is a complex disease of yet unknown pathogenesis. Several factors have been proposed as potential triggers of inflammatory response associated with RA rheumatoid arthritis, including microbes and their products [1, 2]. Products of invading microbes may activate inflammatory cells via Toll-like receptors (TLRs), which leads to immune and inflammatory responses. TLRs activation has been linked to the pathogenesis of rheumatoid arthritis, and both TLR-2 and TLR-4 are potentially critical receptors in the initiation and perpetuation of the inflammatory cycle in arthritis [3]. It has been also demonstrated that endogenous TLR4 ligands (e.g., heat shock proteins and extra domain A fibronectin) are highly expressed in the joints of patients with RA [4–6]. Toll-like receptors are present on tissue synoviocytes but also on peripheral blood monocytes which are recruited to the site of inflammation and are involved in the pathogenesis of synovial inflammation [7–10]. Recent studies described higher expression of TLR4 in leucocytes from patients with ankylosing spondylitis and increased expression of TLR2 and TLR4 receptors in cartilage lesions occurring during OA suggesting that abnormal TLR signaling may also be involved in the pathogenesis of rheumatologic conditions other than rheumatoid arthritis [11, 12].

IL-6 and TNF*α* as well as IL-1 family cytokines and chemokines have been markedly elevated in synovial fluid and synovial membranes from patients with RA, but not in patients with OA [13–15]. Moreover, in sera of RA patients spontaneous IL-1, -6, -8, -18, and TNF*α* production are significantly higher than in healthy individuals and anti-TNF*α* treatment decreases local and systemic expression of TLR2 and TLR4 receptors [16, 17].

Earlier studies documented that peripheral blood mononuclear cells from RA patients generate significantly more inflammatory cytokines and chemokines, although the difference between patients with new onset and established rheumatoid arthritis could not be clearly determined [18–21].

We aimed to assess the effect of Toll-like receptors 2 and 4 stimulation on production of inflammatory cytokines and generation of 15-HETE by PBMCs from patients with newly diagnosed RA in comparison with patients with osteoarthrosis and healthy controls. In addition, we sought to determine in what way the stimulation of TLRs (exemplified by TLR4 stimulation in our experiments) on leucocytes' surface might affect expression of Toll-like receptors themselves on the mRNA level. Finally, we investigated the influence leucocytes' stimulation with mix of strong proinflammatory cytokines on expression of TLR2 and TLR4 at mRNA level.

## 2. METHODS

### 2.1. Patients

Twenty one subjects were recruited to the study, including 7 patients with rheumatoid arthritis (RA), 7 patients with osteoarthrosis (OA) and 7 healthy control (HC) subjects. RA and OA were diagnosed according to American College of Rheumatology guidelines [22]. Severity of rheumatoid arthritis was assessed employing the composite 28-joint disease activity score (DAS28). Although no gold standard for assessment of RA activity exists, the DAS28 score has been adopted by European rheumatologists as a daily practice tool [23, 24]. DAS28 scoring provides a numeric index, in which a score >5.1 implies high-disease activity, a score <3.2 indicates low disease activity, and a score <2.6 indicates remission. Patients with RA were newly diagnosed and had not received disease-modifying antirheumatic drugs (DMARDs) before inclusion into the study and presented with moderate disease activity according to DAS28 (mean index ± SD; 4,31 ± 1,32). Additional parameters used for clinical characterization of patients included anti-cyclic citrullinated peptide antibodies (aCCPs) as well as C-reactive protein (CRP) serum concentration measurements. Serum aCCP presence is a marker of early phase RA. They are more specific as a diagnostic tool than RF and are considered a good predictor of severe erosive disease [25]. CRP elevation is the most useful index of the acute phase response being specific, sensitive, and rising rapidly during 6 to 10 hours after the initiation of an inflammatory process [26].

The aCCPs were detected in all RA patients and in none of patients without RA. The concentration of C-reactive protein (CRP) in serum was increased in RA compared to OA patients. Control group consisted of young healthy volunteers. A clinical characteristic of patients is presented in Table 1.

All participants gave their written consent after being fully informed about the purpose of the study, which was previously approved by Local Bioethics Committee of the Medical University of Lodz.

### 2.2. Purification of peripheral blood mononuclear cells

Blood samples (27 mL per patient) were collected by peripheral venipuncture. Peripheral blood mononuclear cells were purified by centrifugation on Histopaque-1077 (Sigma Aldrich, St. Louis, Mo, USA). Briefly, peripheral blood was diluted with phosphate buffered saline and carefully layered onto Histopaque-1077 and centrifuged at 400 g for 30 minutes at room temperature. The opaque interface was washed three times and finally resuspended at a density of 10^6^ cells/ml in RPMI-1640 medium (Sigma Aldrich, St. Louis, Mo, USA) supplemented with 10% heat inactivated Fetal Bovine Serum (Sigma-Aldrich, St. Louis, Mo, USA), antibiotic (penicillin, streptomycin), and antimycotic (amphothericin B) solution (Sigma Aldrich, St. Louis, Mo, USA).

### 2.3. Cell stimulation

PBMCs were incubated with LPS at a concentration of 1, 10, and 100 ng/mL, biglycan (BGN) at concentrations of 0.1, 1, and 10 ng/mL and with cytokine mix (including 10 ng/mL of IL-1, 10 ng/mL IL-6, and 100 ng/mL TNF*α*) for 24 hours. After centrifugation, supernatants were harvested and analyzed for IL-6 or TNF*α* using ELISA kits (Bender MedSystems Inc., Burlingame, Calif, USA).

### 2.4. RNA isolation and reverse transcription

Total RNA was isolated from leukocytes using Total RNA Mini kits (A&A Biotechnology, Gdynia, Poland) in accordance with manufacturer's instructions. Total RNAs were converted to complementary DNA (cDNA) by random priming with M-MLV reverse transcriptase (Promega, Madison, Wis, USA). After reverse transcription, cDNA was stored at −20°C for further analysis.

### 2.5. Quantitative real-time PCR

Quantitative real-time PCR was performed using ABI Prism 7000 Sequence Detection System (Applied Biosystems, Foster City, Calif, USA). We used iTaq SYBR Green Supermix With ROX (Bio-Rad Laboratories, Inc., Hercules, Calif, USA) as suggested by the manufacturer. In brief, reactions were carried out in 20 *μ*l containing 1 *μ*l cDNA, 10 *μ*l 2 × SYBR Green supermix, and 200 nM of each specific primer. The cycling parameters were 3 minutes at 95°C followed by 45 cycles of denaturation at 95°C for 15 seconds and annealing/extension at 60°C for 45 seconds. For relative quantification, an n-fold differential expression has been calculated using 2^−ΔΔCt^ method. Amplification of each target gene (TLR2 and TLR4) was normalized to that of *β*-actin (housekeeping gene). Amplified products were checked for their correct size by means of agarose gel electrophoresis. Specific primers used for TLR2, TLR4, and *β*-actin amplification were described previously by Gutiérrez-Cañas et al. [27] and their sequences were as follows: TLR2 sense 5′GGA AGA ATC CTC CAA TCA GGC 3′; TLR2 antisense 5′CTT CTG TGA GCC CTG AGG GA 3′; TLR4 sense 5′AGC CAC GCA TTC ACA GGG 3′; TLR4 antisense 5′CAT GGC TGG GAT CAG AGT CC 3′; *β*-actin sense 5′AGA AGG ATT CCT ATG TGG GCG 3′; *β*-actin antisense 5′CAT GTC GTC CCA GTT GGT GAC 3′.

## 3. RESULTS

### 3.1. IL-6 production by PBMCs in response to stimulation with LPS and biglycan

**Figure 1:**
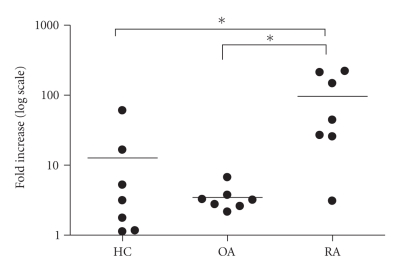
Individual changes in IL-6 concentration in the supernatants of human leucocytes culture after stimulation with LPS at 100 ng/mL; log10 scale; HC-healthy controls, OA-osteoarthrosis, RA-rheumatoid arthritis;*P < .03, Wilcoxon's test.

Nonstimulated PBMCs from RA patients demonstrated a significantly lower production of IL-6 as compared to OA patients (median value 48 times lower) and to control subjects (median value 45 times lower) (Table 2). Stimulation with LPS, an agonist for TLR4 receptors, resulted in significant increase in IL-6 production in all 3 groups. However, in cells from patients with RA, the increase was dramatic (median 30, 48, and 44 folds over baseline after 1, 10, and 100 ng/mL of LPS, resp.) as compared to only moderate (2 to 4 folds) increase observed in cells from patients with OA or healthy subjects (Figure 1).

Incubation of cells with biglycan, the agonist for TLR2 receptors, resulted in small and nonsignificant increase in IL-6 production in both RA patients (after all biglycan concentrations) and AO patients (only after biglycan at 1 ng/mL). In healthy subjects, insignificant increases were observed following 2 highest concentrations, and small decrease (22%) was noticed after the lowest biglycan concentration.

### 3.2. TNF*α* production by PBMCs in response to stimulation with LPS and biglycan

**Figure 2:**
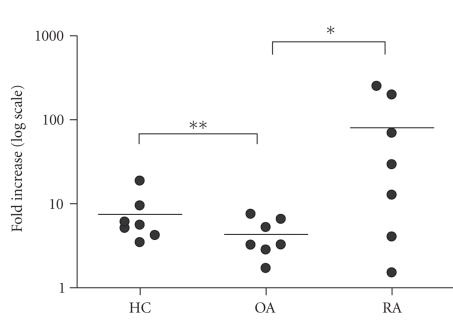
Individual changes in TNF*α* concentration in the supernatants of human leucocytes culture after stimulation with LPS 100 ng/mL; log_10_ scale; HC-healthy controls; OA-osteoarthritis; RA-rheumatoid arthritis;*p = .04; **p = .027.

Nonstimulated PBMCs from RA patients generated significantly less TNF*α* as compared to OA patients (mean 12 times less) and control subjects (mean 10 times less) (Table 3). Incubation with LPS induced an increase in TNF-*α* production in all 3 groups. However, similarly to IL-6, the increase in TNF*α* production was significantly more intense after LPS at 10 and 100 ng/mL (resp., 31 and 29 folds over the baseline) as compared to that observed in cells from patients with OA (mean 3 to 8 folds increase for different LPS concentrations) or healthy subjects (mean 4- to 7-fold increase for different LPS concentrations) (Figure 2). Stimulation with biglycan induced a significant increase in TNF*α* production only in PBMCs from healthy subjects in response to the highest concentration.

### 3.3. 15-HETE generation by PBMCs in response to stimulation with LPS and biglycan

**Figure 3:**
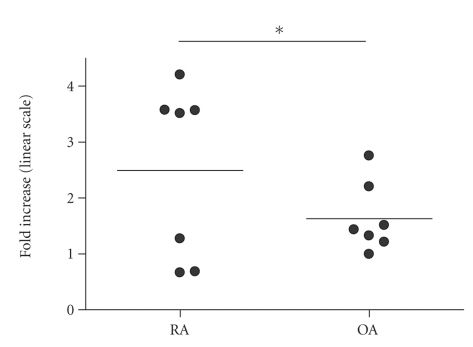
Changes in 15-HETE release into mononuclear cells supernatants after stimulation with 100 ng/mL LPS in individual patients OA-osteoarthritis; RA-rheumatoid arthritis;*p < .01.

15-HETE was generated by unstimulated PBMCs cells from both RA and OA patients in similar amounts. Stimulation with LPS increased 15-HETE generation in 5 RA patients (range 27–320%) while decreased in 2 patients (32% and 34%, resp.). In OA patients, LPS induced a significant (*P* < .005) increase in 15-HETE production (mean increase 43%; range 20–175%) (Figure 3). Incubation with biglycan did not affect 15-HETE generation.

### 3.4. Effect of cytokines and LPS on TLR2 and TLR4 gene expression in PBMCs

Expression of TLR2 mRNA in nonstimulated, cytokine mix-stimulated, or LPS stimulated cells was similar in RA, OA and HC subjects (Table 4(a)). Mean expression of TLR-4 mRNA in nonstimulated as well as in LPS or cytokine-mix stimulated cells was similar in patients with RA and OA, although in both groups, it was significantly lower than in cells from HC.

Incubation with cytokine mix tended to decrease both TLR2 and TLR4 expressions although the decrease was significant for both receptors in OA patients and for TLR4 in HC subjects (Table 4(b)). TLR2 gene expression was significantly increased by LPS in healthy controls, while significant suppression of TL4 mRNA expression was observed after LPS in cells from patients with OA.

## 4. DISCUSSION

This study was aimed at investigation of the significance of TLRs and their stimulation in contribution to development of a chronic inflammatory process present in patients with rheumatoid arthritis. We have demonstrated that peripheral blood mononuclear cells from patients with newly detected RA spontaneously generated significantly smaller amounts of interleukin-6 and TNF*α* as compared to monocytes from OA patients or healthy subjects and that stimulation with LPS resulted in dramatically higher augmentation of cytokine production in cells from RA patients as compared to OA patients. Low-basal production of cytokines is an unexpected finding, since these two cytokines are considered to play a significant role in the pathogenesis of joint inflammation in RA; TNF*α* and IL-6 may stimulate collagenase production, increase bone resorption and inhibit cartilage regeneration targeting fibroblasts, osteoclasts, and chondrocytes [28, 29]. Accordingly, previous studies demonstrated increased serum concentrations and enhanced generation of both cytokines by mononuclear cells from patients with RA [17–21]. Low baseline cytokine production in circulating mononuclear cells may be explained by the fact that we have studied patients with early detected RA; it is possible that at this stage of the disease, cells with high potential for cytokine production have been recruited to the site of inflammation (synovium) and only less active ones are available in the peripheral circulation. The most striking observation is a dramatic difference in response to stimulation with lipopolysaccharides between mononuclear cells from RA as compared to OA and healthy controls. Although in patients with OA and healthy controls LPS induced significant (2-7-fold) increase in production of both cytokines, the increases in monocytes from RA patients were dramatic: 17 to 31 folds for TNF*α* and 30 to 48 folds for IL-6. These observations indicate that monocytes from patients with early detected RA, in spite of having low-baseline production of cytokines demonstrate high potential for cytokine generation upon stimulation with LPS. LPS is thought to activate cells through specific TLR4 receptor which triggers a complex signaling cascade leading to release of inflammatory mediators potentially contributing to development of synovial inflammation. It has been previously demonstrated that TLR2 and TLR4 ligands enhance cytokine expression in synovial fluid macrophages from RA patients and induce catabolic responses in chondrocytes from OA patients [12, 30]. Our observation is in line with putative role of TLR4 receptors in the development of inflammatory responses in rheumatoid arthritis, suggesting that even in patients with newly detected and not yet pharmacologically treated RA, circulating inflammatory cells (monocytes) can be easily triggered via TLR4 stimulation to generate proinflammatory cytokines.

15-HETE is an arachidonic acid metabolite generated by reticulocyte-type B synoviocytes in human rheumatoid arthritis with potential modulatory effect on inflammation [31, 32]. We have demonstrated for the first time that stimulation of TLR4 but not TLR2 receptors increases generation of 15-HETE by mononuclear cells from both RA and OA patients. Although LPS consistently increases 15-HETE generation in cells from 7 of 7 OA patients, in 4 of 7 patients with RA, the enhancement in 15-HETE generation by LPS was much higher. Further studies are necessary to elucidate the potential role of TLR4-triggered 15-HETE generation in the pathogenesis of RA and OA.

Higher responsiveness of monocytes from RA subjects to LPS as compared to cells from OA subjects and healthy controls could be explained by increased expression of TLR4 receptors on cell surface. Increased expression of Toll-like receptors on synoviocytes derived from the site of rheumatoid inflammation has been reported [33, 34]. However, information on TLRs expression on peripheral blood monocytes is less consistent and studies using either cytofluorometry to detect TLR2 and TLR4 protein or RT-PCR to detect receptor transcript reported variable results [29, 30].

In this study, we used quantitative real-time PCR technique to asses TLR2 and TLR4 expressions in mononuclear cells. Expression of those 2 types of TLRs was investigated in nonstimulated cells as well as under stimulatory conditions. We have chosen LPS—a TLR4 agonist—and mix of potent proinflammatory cytokines as stimulants for this part of our experiments. Although the level of expression of TLR2 gene was not significantly different among three groups, expression of TLR4 mRNA was significantly decreased in cells from RA or OA patients as compared of healthy controls. However, these data have to be interpreted with caution since mRNA was isolated from total leukocyte population and its measurement might not reflect receptor expression in monocytes, which are primarily targeted by TLR agonists. Moreover, recent studies demonstrated dissociation of mRNA expression of Toll-like receptors from the modulation of TLR-mediated responses, suggesting that factors other than receptor expression may be responsible for increased responsiveness of RA monocytes to TLR ligands [26, 35]. RT-PCR technique allowed us to analyze TLR mRNA expression in response to stimulation with cytokine mix (IL1, TNF*α* and IL-6) and LPS. While cytokine mix tended to decrease Toll-like receptors gene expression in all groups studied, LPS had more variable effects: enhancement of TLR2 receptors was observed in healthy controls while significant depression of TLR4 expression was noticed in patients with osteoarthritis. These observations confirm that TLR agonist similarly to endogenous cytokines may modulate expression of Toll-like receptors on leukocytes and suggest that this modulation may vary in cells derived from patients with different form of rheumatic pathology or from healthy persons.

In conclusion, our study demonstrated a significant overresponsiveness of peripheral blood mononuclear cells from patients with recently diagnosed rheumatoid arthritis to stimulation with a bacterial product, lipopolysaccharide. Regulation of TLR expression by cytokine and TLR agonist may differ in healthy subjects and patients with arthritides.

## Figures and Tables

**Table 1:** Clinical characteristic of patients.

	Rheumatoid arthritis	Osteoarthrosis	Healthy control
Female/male	6 /1	7/0	6/1
Age in years (median; range)	56 (35–68)	59 (53–75)	32 (27–55)
aCCP in IU/mL (mean ± SD)	68,76 ± 47,17	4,02 ± 5,79	Not done
CRP mg/l (mean ± SD)	12 ± 6	6 ± 0	Not done
DAS28 (median; min-max)	4,2 (1,96–6,27)	n/a	n/a

**Table 2:** Spontaneous and stimulated IL-6 production by peripheral blood mononuclear cells from patients with rheumatoid arthritis (RA), osteoarthrosis(OA), and healthy controls (HC); median values; *P* < .05*—significantly different from nonstimulated; #—significant difference between RA and HC ^∧^—significant difference between RA and OA.

	Nonstimulated	Stimulus					
		LPS 1 ng/mL	LPS 10 ng/mL	LPS 100 ng/mL	BGN 0,1 ng/mL	BGN1# ng/mL	BGN#10 ng/mL
Group of subjects	concentration of IL-6 (ng/mL)						

RA	0,56#** ^∧^	22,87***	42,95***^∧^	46,72***	2,0** ^∧^	1,24#** ^∧^	1,55#** ^∧^
OA	27,2	43,6***	88,47***	75,1***	22,6	33,01	23,0
HC	25,45	86,46***	73,47	99,11***	21,15***	50,81	46,0

**Table 3:** Spontaneous and stimulated TNF*α* production by peripheral blood mononuclear cells from patients with rheumatoid arthritis (RA), osteoarthrosis (OA), and healthy control (HC); *P* < .05*—significantly different from nonstimulated; #—significant difference between RA and HC ^∧^—significant difference between RA and OA.

	Nonstimulated	LPS 1 ng/mL	LPS 10 ng/mL	LPS 100 ng/mL	BGN 0,1 ng/mL	BGN 1 ng/mL	BGN 10 ng/mL
Group of subjects	Concentration of TNF*α* (ng/mL)						

RA	0,05^∧^#	0,72^∧^#	2,02*	2,84*	0,1^∧^#	0,26	0,07^∧^#
OA	0,59	4,71***	2,48***	2,62***	0,84	0,45	0,71
HC	0,49	2,59***	1,98***	1,94***	0,63	0,65	0,68***

**Table:** (a) Target gene expression is normalized to housekeeping gene expression and presented as *n*-fold of the expression in HC group (2^−ΔΔCT^). ***denotes significant difference in expression comparing with HC (*P* < .03).

	Nonstimulated	Cytokine mix	LPS
TLR2			

HC	1	1	1
RA	2.045	2.915	0.036
OA	1.218	1.66	0.074

TLR4			

HC	1	1	1
RA	0.008***	0.225	0.0009***
OA	0.004***	0.044***	0.00002***

**Table:** (b) Target gene expression after stimulation with cytokine mix or LPS is normalized to housekeeping gene expression and presented as *n*-fold of the expression in non stimulated cells from patients from every group (2^−ΔΔCT^). Significant differences in expression compared to nonstimulated cells are marked with*P < 0.02; ^#^ *P* < 0.01.

	Nonstimulated	Cytokine mix	LPS
TLR2			

HC	1	0.268	17.753***
RA	1	0.382	0.316
OA	1	0.365** ^#^	1.071

TLR4			

HC	1	0.00006***	0.1
RA	1	0.212	1.498
OA	1	0.093** ^#^	0.092***
